# Capacitive Coulometric Readout of Polyaniline Membrane-Based pH Sensors in Combination with Cyclic Voltammetry and Electrochemical Impedance Spectroscopy

**DOI:** 10.3390/membranes15100320

**Published:** 2025-10-17

**Authors:** Tingting Han, Tao Song, Dongxue Han, Li Niu

**Affiliations:** 1 Guangzhou Key Laboratory of Sensing Materials & Devices, Center for Advanced Analytical Science, School of Chemistry and Chemical Engineering, Guangzhou University, Guangzhou 510006, China; dxhan@gzhu.edu.cn (D.H.); lniu@gzhu.edu.cn (L.N.); 2 State Key Laboratory of Pulp and Paper Engineering, South China University of Technology, Guangzhou 510640, China; songt@scut.edu.cn; 3 School of Chemical Engineering and Technology, Sun Yat-sen University, Zhuhai 519082, China

**Keywords:** constant potential coulometry, potentiometry, PANI membrane growth, solid-contact ion-selective electrodes, electrochemical impedance spectroscopy, cyclic voltammetry

## Abstract

In this study, a polyaniline (PANI)-based solid-contact pH sensor was fabricated, and its amperometric and coulometric response was investigated both without and in series with capacitors (10 and 47 µF). The conducting polymer PANI membrane was electropolymerized on the electrode surface to serve as an ion-to-electron transducer. The amperometric and coulometric performance of the PANI-based sensor in series with a capacitor (10 µF) was reduced to the order of seconds, and the cumulated charge *Q* was standardized, significantly minimizing the influence of applied potential. Electrochemical impedance spectroscopy, constant potential coulometry, and cyclic voltammetry demonstrated that a larger low-frequency capacitance corresponds to a greater cumulated charge, reflecting the doping level of the electropolymerized PANI membrane. The growth of the PANI membrane, represented by charge *Q*, increased exponentially with the number of polymerization cycles, following a power-law relationship with exponents (α) of 2.14 (1–25 cycles) and 2.97 (30–100 cycles), consistent with a transition from a layered (10 cycles) to a porous morphology (50 cycles). Furthermore, a linear dependence of cumulated charge *Q* on pH was observed, demonstrating that capacitive coulometric readout offers a promising and practical approach for wearable ion sensors.

## 1. Introduction

Ion-selective electrodes (ISEs) have found extensive application across diverse fields, such as clinical analysis, food safety, environmental monitoring, and healthcare [1,2,3,4,5,6]. With the increasing emphasis on health surveillance, there is a corresponding demand for enhanced sensor sensitivity, particularly in clinical diagnostics and wearable technology [3,4]. Wearable sensors function as non-invasive tools for quantifying ions or organic molecules that serve as biomarkers of physiological status [7,8].

Conventional potentiometry relies on measuring the equilibrium potential difference across a selective membrane, which is logarithmically related to the activity of the target ion following the Nernst equation. This logarithmic relationship imposes an intrinsic sensitivity barrier. For a monovalent ion like H^+^, a theoretical potential change of approximately 59 mV corresponds to a ten-fold change in activity of 1 pH unit. Consequently, detecting a minute change of 0.01 pH units requires a reliable measurement of a potential shift of just 0.59 mV [2,9]. In practical scenarios, consistently achieving this level of resolution is challenging due to signal drift, reference electrode instability, and electrical noise, particularly in complex matrices like blood or biofluids [1,10]. This sensitivity limitation restricts the ability of even the most advanced ISEs to detect subtle but physiologically or environmentally critical fluctuations [1,7,11].

The ongoing research on high-performance pH sensing has driven significant advancements in detection methodologies. We have witnessed the emergence of diverse sensing strategies, each offering distinct advantages: Utilizing an ionic liquid salt bridge functionalized reference electrode enables us to accurately determine pH values within 0.03 pH units and have a shorter response time in low ionic strength pH aqueous solution [12]. Employment of metal oxides and nanomaterials has been developed to increase the capacitance, resulting in high potential stability to decrease the potential drift and minimization of the water layer effect [13,14]. By virtue of their optical transduction mechanism, these sensors are not only immune to electromagnetic interference but also achieve high sensitivity and superior long-term stability [15,16]. Highly sensitive amperometric sensors capable of resolving changes as minute as responses to below 0.01 pH units have been realized [17,18] alongside innovative platforms like organic electrochemical transistors (OECTs) with designs capable of resolving pH shifts [19]. Furthermore, regarding technological progress, miniaturized potentiometric/conductometric sensors for lab-on-a-chip applications and the integration of smart materials and IoT-enabled platforms are paving the way for next-generation, intelligent sensing systems [7,8].

Among those diverse developments, constant potential coulometry emerged as a fundamentally different transduction mechanism, utilizing cumulated charge as the analytical signal [20,21,22,23]:

(1) $$Q=C_{cp}\frac{0.0592}{z_{i}}log\left(\frac{a_{i}\left(initial\right)}{a_{i}\left(final\right)}\right)$$

where *Q* is the cumulated charge signal of constant potential coulometry, *C_cp_* is the capacitance of the conducting polymer, *z_i_* is the charge of the primary ion *i*, and *a_i_*(*initial*) is the activity of the primary ion before the activity change. *a_i_*(*final*) is the activity of the primary ion after the activity change [22]. The key advantage is signal amplification; the cumulated charge is directly proportional to the change in ion activity and can be enhanced by increasing the capacitance of the solid-contact layer [23]. The coulometric response of SCISEs demonstrated exceptionally high sensitivity for ions like K^+^ and Ca^2+^, detecting activity changes as low as 0.1% [24]. A significant innovation within this framework involves utilizing ion-selective electrodes as reference electrodes [25,26] and connecting an external capacitor in series with the SCISE, which has been shown to drastically reduce the response time and further improve signal-to-noise ratios, enabling sensitivity down to 0.001 pH units in seawater [27]. A two-electrode protocol that eliminates current flow between the counter and reference electrodes enabled the detection of concentration changes as low as 0.026% [28]. Furthermore, the application of coulometric transduction has been successfully extended to flexible sensor platforms, highlighting its potential for wearable applications [29].

In this work, the amperometric and coulometric responses of the PANI-based pH sensors without a capacitor, and in series with 10 µF or 47 µF capacitors, were investigated. The performance of the PANI-based pH sensor was characterized in Tris-buffer, with pH changes induced by the addition of NaOH. The correlation between the low-frequency capacitance (from EIS), voltametric charge, and the constant potential coulometric readout was analyzed. The growth of the PANI film via cyclic voltammetry was examined, and its structural evolution (from layers to porous networks) was in good agreement with its electrochemical function. Finally, the coulometric performance of the PANI-based pH sensor was evaluated in a flexible configuration.

## 2. Experimental Section

### 2.1. Reagents and Chemicals

Aniline (97%) and tetrahydrofuran (THF) were purchased from Sigma-Aldrich (Saint Louis, MO, USA), and were of Selectophore purity grade. Hydrochloric acid (HCl) (≥99.5%) and sodium hydroxide (NaOH) (≥99.5%) were purchased from Guangzhou Chemical Company (Guangzhou, China). Phosphorous acid (97%), acetic acid (≥99%), and boric acid (99%) were obtained from Beijing Innochem Company (Beijing, China). Aluminum oxide (Al_2_O_3_) powder (0.3 μm) was purchased from Guangdong Liquefied Air Co., Ltd. (Qingyuan, China). De-ionized water (resistivity > 18.2 MΩ·cm) was used for all the experiments, which was produced using de-ionized water system from Sichuan Water purifier Instrument Co., Ltd. (Chengdu, China).

### 2.2. Electrode Preparation of PANI-Based pH Sensor

Glassy carbon (GC) electrode (Փ_GC_ = 3 mm) wrapped in PVC body (Փ_PVC_ = 8 mm) was first polished with diamond paste of 1 µm and 0.3 µm Al_2_O_3_ powder. Then, the well-polished electrodes were ultrasonicated for 5 min in ethanol and water baths, respectively. The electropolymerization was performed in a three-electrode electrochemical cell, and a commercial Ag/AgCl/3 M KCl/1 M LiAc was used as the reference electrode and a Pt electrode as the counter electrode, in connection with a CHI 1030C electrochemical workstation (Shanghai Chenhua Apparatus Corporation, Shanghai, China). Prior to polymerization, clean and well-polished GC electrodes were prepared. Polymerization solution with 0.1 M aniline and 1.0 M HCl was first purged with N_2_ gas for ca. 10–15 min to remove O_2_ from the aqueous solution. Polyaniline (PANI) was electropolymerized on the GC surface, utilizing cyclic voltammetry with a potential window from −0.2 V to 0.82 V and a scan rate of 50 mV/s for 25, 50, and 100 cycles [30]. The obtained solid-contact membrane was referred to as polyaniline doped with Cl^−^ anion PANI/GC (Փ_GC_ = 3 mm) was conditioned in 1 mM HCl before measurement.

### 2.3. Potentiometric Calibration of PANI-Based pH Sensor

The potentiometric calibration of the PANI-based pH sensor was performed with EMF 16 (Lawson Lab, Inc., Irvine, CA, USA). Potentiometric measurement of the PANI-based SCISEs with 25, 50, and 100 cycles was performed to verify the proper function of the SCISEs in Tris-buffer by adding 0.2 M NaOH solution. The pH aqueous solution was prepared with a starting solution of Tris-buffer by adding a certain volume of 0.2 M NaOH to change the pH, and the pH changes in the aqueous solution were checked with a pH meter(DZS-708T, Lei-ci, Shanghai, China). The potentiometric measurement of the PANI-based pH sensor was performed following the identical experimental process. The pH changes for each step were ca. 0.5 decades, and each potential step measurement took ca. 2 min, and the calibration pH range was from 5.13 to 8.27.

### 2.4. Electrochemical Impedance Spectroscopy and Morphology Characterization of PANI-Based GC Electrodes

Electrochemical impedance spectroscopy (EIS) measurements for PANI/GC electrodes with 25, 50, and 100 cycles were performed in 0.1 M HCl with variable applied potentials, i.e., open circuit potential (OCP), 0 V, 0.1 V, 0.15 V, and 0.2 V, using the Gamry reference 600 plus electrochemical workstations (Gamry Instruments, Warminster, PA, USA) in a three-electrode electrochemical cell. A single junction Ag/AgCl/3 M KCl was used as the reference electrode and a platinum wire as the counter electrode. The frequency range was from 1 MHz to 10 mHz with an excitation amplitude of 10 mV (RMS).

Scanning electron microscopy (SEM) using a Phenom Nano SEM (Phenom Scientific, Eindhoven, Holland) at 10 KV were carried out to analyze the morphology of PANI/GC electrode with 10 and 50 polymerization cycles.

### 2.5. Chronoamperometric and Coulometric Response of PANI-Based GC Electrode

The chronoamperometric and coulometric responses of the PANI/GC electrodes were performed using the CHI 1030C electrochemical workstation (Shanghang CHI Apparatus Corporation, Shanghai, China). The chronoamperometric responses of the PANI/GC electrode (Փ_GC_ = 3 mm) with 25, 50, and 100 cycles were performed with a Tris-buffer solution by adding 0.2 M NaOH with pH changes at 0.5 decades/step at open circuit potential.

The amperometric and coulometric responses of the PANI/GC electrodes with 25, 50, and 100 cycles were performed to be first stabilized to reach equilibrium at open circuit potential with Tris-buffer +0.2 M NaOH as the starting aqueous solution. The addition volume of 0.2 M NaOH in the starting solution was adjusted based on the required starting pH. Then, the volume of 0.2 M NaOH was added to change the pH. The time intervals between each addition were 30 s–2.5 min. Each addition of 0.2 M NaOH with a volume of 1 mL and 0.5 mL into the starting Tris-buffer +0.2 M NaOH aqueous solution resulted in ΔpH ≈ 0.5 decades/step and ΔpH ≈ 0.25 decades/step, respectively. The amperometric and coulometric responses of the PANI-based pH sensors with 50 and 100 cycles were performed in series with capacitors 10 µF and 47 µF, following the identical experimental process. The time intervals were ca. 30 s between each step. The amperometric and coulometric responses of the PANI-based GC electrodes with 50 cycles were also performed with variable applied potentials, OCP, 0 V, and 0.2 V in the case of not having a capacitor, and in series with a capacitor of 10 µF and 47 µF.

The chronoamperometric and coulometric reversible test of the PANI-based pH sensor with 50 cycles in connection with capacitors 10 µF and 47 µF was performed with a starting solution pH of Tris-buffer +0.2 M NaOH at 7 by adding a volume of 0.2 M NaOH to increase pH to 7.5 manually, and following that, the addition of certain volume of Tris-buffer solution results in pH back to 7. The pH changes in the aqueous solution were switched between 7 and 7.5.

### 2.6. Flexible PANI-Based pH Sensor Preparation

A poly(ethylene terephthalate) (PET) substrate (Guangzhou Chemical Company, Guangzhou, China) of 7 × 7 cm^2^ was first cleaned successively with water, acetone, and isopropanol under ultrasonication, and then it was etched with O_2_ plasma for 2 min. Then, the microwell pattern of the Au electrode was fabricated by magnetron sputtering deposition with a Au target at a power of 60 W for 25 min in a Ar atmosphere by using ultra-high vacuum sputtering technology (AJA Orion 5, Scituate, MA, USA). After coating with a polydimethylsiloxane (PDMS) insulating layer, the flexible electrode was dried at 90 °C for 50 min. The flexible pH sensors (Փ_AU_ = 5 mm) were obtained by electropolymerizing PANI with 50 cycles on a PET substrate sputtered with gold. The polymerization was performed in 1.0 M HCl and 0.1 M aniline electrolyte with a 50 mV/s scan rate. The potential window is from −0.2 V to 0.82 V. After rinsing with de-ionized water and drying for at least 6 h, the flexible pH sensors were conditioned in 1 mM HCl solution overnight.

### 2.7. Coulometric Response of Flexible PANI-Based pH Sensors in Series with Capacitor

Potentiometric measurement of the flexible PANI-based SCISEs with 50 cycles was performed to verify the proper function of the SCISEs in Tris-buffer +0.2 M NaOH by adding 0.2 M NaOH solution manually. The pH changes in the Tris-buffer +0.2 M NaOH with standard addition of 0.2 M NaOH were performed following the experimental process described above. The chronoamperometric and coulometric responses of the flexible PANI-based pH sensor in series with a capacitor, 10 µF and 47 µF, were performed with pH changes at 0.5 decades/step, and the pH calibration range is 7–8.

## 3. Results

### 3.1. Amperometric and Coulometric Response of PANI-Based pH Sensor in Series with Capacitor

**Figure 1:**
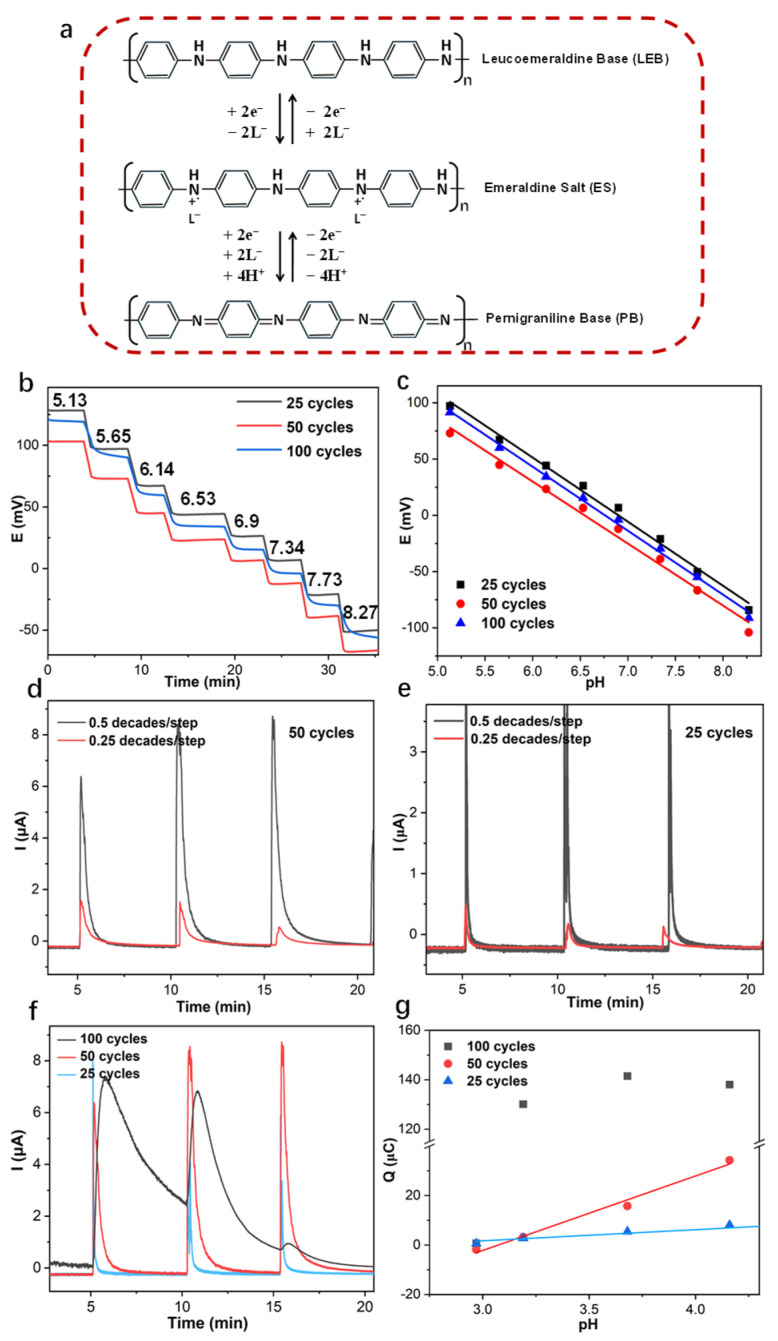
(**a**) The ion-to-electron transfer mechanisms of PANI solid contact; potentiometric measurement (**b**) and calibration (**c**) of PANI-based pH sensor with pH changes at 0.5 decades/step; (**d**) amperometric and (**e**) coulometric response of PANI-based pH sensor with 50 cycles under pH changes of 0.5 and 0.25 decades/step. (**f**) amperometric and (**g**) coulometric response of PANI-based pH sensors with 25, 50, and 100 polymerization cycles; The measurements were conducted in a Tris-buffer solution containing 0.2 M NaOH. pH adjustments were made by adding specific volumes of 0.2 M NaOH.

PANI solid contact was electropolymerized on the GC electrode surface, utilizing cyclic voltammetry (Figure S1) [30]. The ion-to-electron transfer mechanism of the PANI solid contact was shown in Figure 1a. The potentiometric measurement of the PANI-based pH sensor was performed with Tris-buffer +0.2 M NaOH by adding a certain volume of 0.2 M NaOH manually, resulting in pH changes from 5.13 to 8.27. The calibration slope of the PANI-based GC electrode at 55 ± 2 mV/decade proves the proper function of the electrode (Figure 1b,c). In the case of utilizing the PANI solid contact as ion-to-electron transducers (Figure 1a), as the pH of the aqueous solution changes, the doping/undoping counter ions would occur on the solid-contact film PANI. The state of the PANI solid contact changed between the reduced non-conductive form leucoemeraldine base (LEB), in the conductive form, and emeralidine salt (ES). For the coulometric response of the PANI-based pH sensor, when the pH changes, the potential of the PANI-based pH sensor changes, and the current flow between the working electrode (PANI-based sensor) and the counter electrode Pt to balance the potential changes due to the pH changes in the aqueous solution. The integral of current with time results in cumulated charge *Q*. The cumulated charge *Q* analytical signal was investigated. While the PANI-based pH sensor was in series with the capacitor, the cumulated charge signal response would be mainly dominated by the capacitor 10 μF/47 μF [27].

The peak current (*i_p_*) is found at *t*_0_ as follows:

(2) $${\;i}_{p}=\frac{s}{z_{i}R_{\mathrm{cell}}}\log\left(\frac{a_{i}\left(\mathrm{initial}\right)}{a_{i}\left(\mathrm{final}\right)}\right)$$

where *R_cell_* is the resistance of the electrochemical cell, *s* is 59.2 mV for 25 °C, and *z_i_* is the charge of the analyte ion. The peak current of the amperometric response for the PANI-based pH sensor is linearly proportional to the resistance of the solid-contact film and is also closely related to the activity change of the primary ion. As the pH sensor has an identical thickness to PANI solid contact, the current peak of the pH sensor gives a larger current, with 0.5 decades/step instead of 0.25 decades/step for both the PANI-based sensors with 25 and 50 cycles (Figure 1d,e). The peak current of the PANI-based pH sensor with 50 cycles is larger than the solid-contact film with 25 cycles (Figure 1d), which is in good agreement with the resistance results of the electrochemical impedance spectrum for the PANI-based pH sensor with 25 and 50 cycles (Table S2).

The amperometric and coulometric responses of the PANI-based pH sensors prepared with 25, 50, and 100 polymerization cycles are shown in Figure 1f,g. As the thickness of the PANI membrane increased from 25 to 100 cycles, the required equilibration time extended from 30 s to over 5 min. The linear slope of cumulated charge *Q* versus pH change increased from 2.49 μC/pH for 25 cycles to 30.13 μC/pH for 50 cycles.

The coulometric response of the PANI-based transducer is intrinsically governed by its complex redox chemistry and the kinetics of the accompanying ion-exchange processes. Upon a pH change in the solution, the initial sensing mechanism involves the oxidation of the non-conductive LEB state to the conductive ES form. This transition is central to the signal generation, as it involves the insertion of anions (e.g., Cl^−^) into the polymer matrix to maintain electroneutrality, thereby contributing to the measured Faradaic (redox) charge. However, our results indicate that for the thick PANI film (100 cycles), the system fails to return to its initial equilibrium state within the 5 min timeframe (Figure 1f). This kinetic limitation is critical. The subsequent potential step drives a portion of the PANI film beyond the conductive ES state to the fully oxidized, highly resistive pernigraniline base (PB) form. This PB state is electrochemically irreversible under these conditions and acts as a passivating layer, severely impeding both electron transfer and ion transport. Consequently, the charge involved in this undesirable side reaction is not recoverable, leading to the observed signal loss from the second pH step onward.

**Figure 2:**
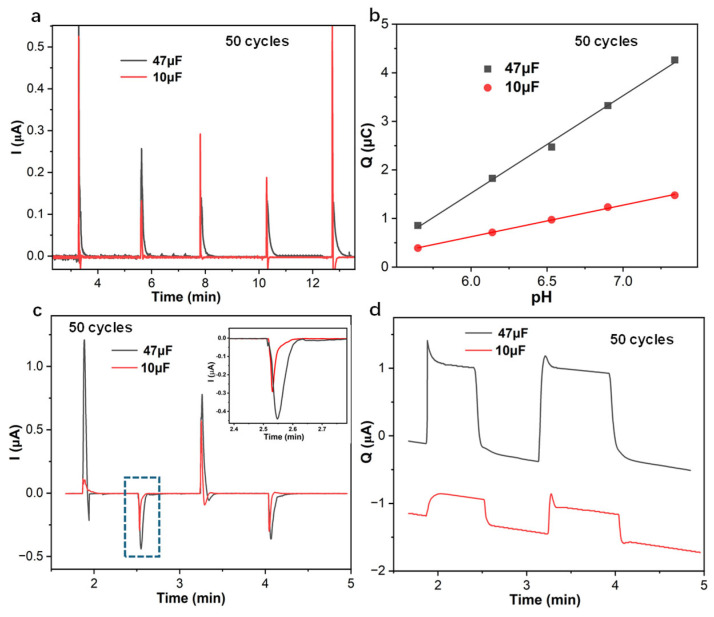
(**a**) Amperometric and (**b**) coulometric response of PANI-based pH sensor with 50 cycles in series with capacitors 10 μF and 47 μF with pH changes at 0.5 decades/step; Reversible (**c**) amperometric and (**d**) coulometric response of PANI-based pH sensor with 50 cycles in series with capacitors (10 μF, 47 μF), while the pH of electrolyte switches between 7 and 7.5.

Once the PANI-based pH sensor with 50 cycles, as a working electrode, was in series with capacitors 47 μF and 10 μF, the amperometric response of the PANI-based pH sensor decreased to less than 5 s with the pH change at 0.5 decades/step and the pH range from 5.3 to 7.8 (Figure 2a). While the PANI-based pH sensor was in series with the capacitor (Figure 2b), the linear slope of cumulated charge *Q* with respect to pH changes was at 2 μC/pH for the electrode in series with 47 μF and 0.69 μC/pH for 10 μF. The linear slope increased as the connected capacitor increased from 10 μF to 47 μF. Also, the following, according to Kirchhoff’s law, is true [27]:

(3) $$\frac{1}{C_{total}}=\frac{1}{C_{PANI}}+\frac{1}{C_{capacitor}}$$

The resulting capacitance is dominated by the connected capacitor, which is magnitudes smaller than the capacitance 1065 μF of the PANI-based pH sensor with 50 cycles, calculated from the electrochemical impedance spectrum at low frequency 10 mHz (Table S2). The external capacitors 10 μF and 47 μF are beneficial for the fast response (seconds equilibrium time) of the PANI-based pH sensor.

The amperometric and coulometric reversible responses of the PANI-based pH sensor with 50 cycles were in series with 10 μF and 47 μF, while pH switches between 7 and 7.5 are shown in Figure 2c,d. The reversible amperometric response of the PANI-based pH sensor shows that the equilibrium of the doping/undoping PANI reaction can be reached in 3 s with 0.5 decade pH changes. When the PANI-based pH sensor was in series with 47 μF, the resulting charge *Q* was 1.16 μF, while the corresponding charge was 0.37 μF for 10 μF. With the pH changes switching between 7 and 7.5, the resulting identical charge *Q* of the PANI-based pH sensor implies that the PANI solid contact doping/undoping counter ion Cl^−^ is a reversible process, with the conditioned 1 mM Cl^−^ counter ions in the PANI solid-contact film. The drifting of the cumulated charge over time is probably due to the potential drifting of the PANI-based electrodes. While the PANI-based pH sensor was in series with capacitors 10 μF and 47 μF, the precision of the cumulated charge *Q* can be improved in favor of response time, decreasing it by seconds, and, in a way, this decreases the potential drifting impact on the results of the cumulated charge *Q* [26,28].

### 3.2. EIS Low-Frequency Capacitance, Current, and Charge of CV, and Coulometric Response of PANI-Based pH Sensor with Variable Applied Potentials

**Figure 3:**
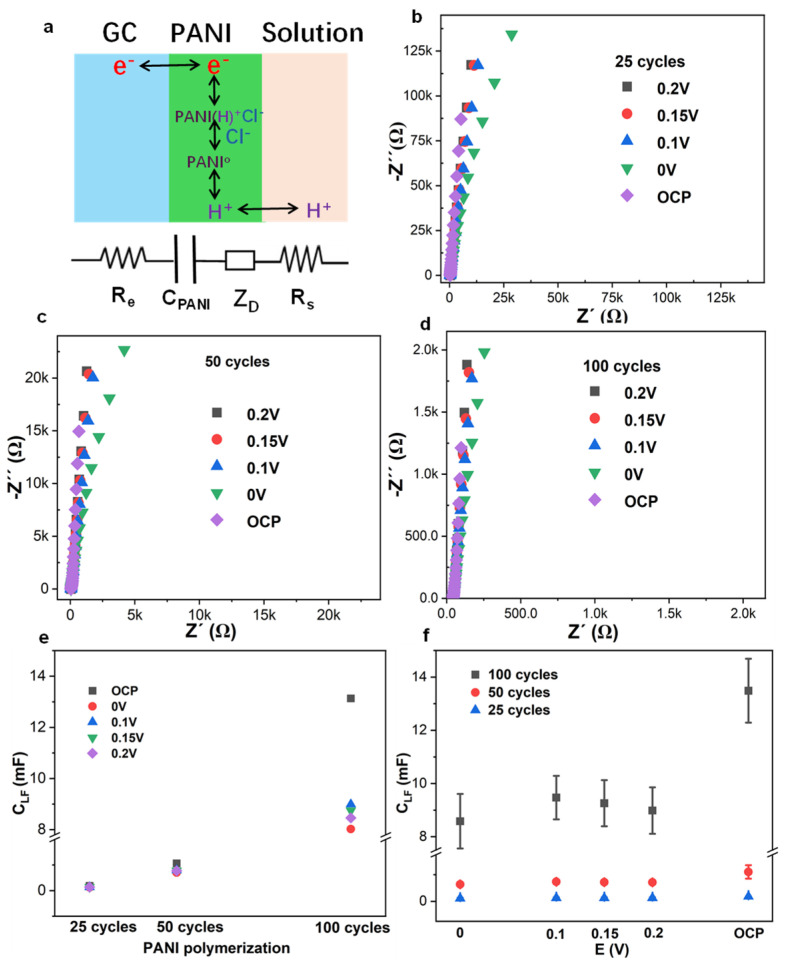
(**a**) Schematic picture of ion-to-electron transfer mechanisms of the PANI-based GC electrode and the corresponding equivalent circuits; electrochemical impedance spectrum of the PANI-based GC electrode with (**b**) 25, (**c**) 50, and (**d**) 100 cycles with applied potentials at open circuit potential (OCP), 0 V, 0.1 V, 0.15 V, and 0.2 V in 0.1 M HCl aqueous solution; (**e**) the corresponding EIS low-frequency capacitance *C_LF_* of the PANI-based GC electrode with 25, 50, and 100 cycles determined at 10 mHz; (**f**) EIS low-frequency capacitance *C_LF_* (10 mHz) of the PANI-based GC electrode with 25, 50, and 100 cycles vs. the applied potentials.

As shown in Figure 3a, the PANI solid contact acted as an ion-to-electron transducer in addition to the corresponding equivalent circuits of the PANI-based GC electrodes [31,32]. The electrochemical impedance spectrum of PANI-based GC electrodes with 25, 50, and 100 cycles was applied with variable potentials at OCP, 0 V, 0.1 V, 0.15 V, and 0.2 V in 0.1 M HCl with frequency conversion from 1 MHz to 10 mHz (Figure 3b–d). The low frequency of the electrochemical impedance spectrum for the PANI-based pH sensor with 25, 50, and 100 cycles gave a nearly 90° tail, behaving as a capacitor (*C_PANI_*) [33]. The low-frequency capacitance (*C_LF_*) at 10 mHz of the PANI-based pH sensor in 0.1 M HCl can be calculated according to the equation *C_LF_* = −1/(2*πfZ″*), where *f* is 10 mHz, and *Z’’* represents the imaginary value of the spectrum at 10 mHz. The impedance real value *Z’* represents the resistance of the PANI solid contact at the semicircle high frequency. The PANI solid-contact resistance *Z’* and low-frequency capacitance *C_LF_* determined at 10 mHz of PANI-based pH sensors were calculated and listed in Tables S1 and S2.

**Figure 4:**
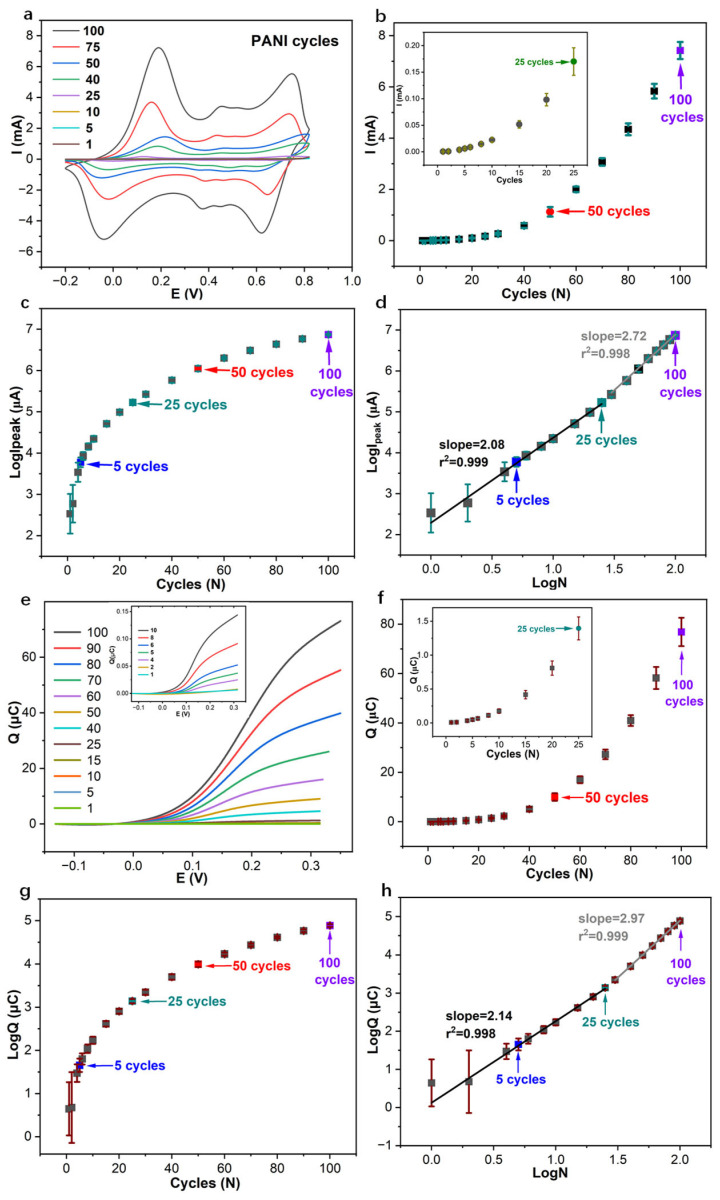
(**a**) Cyclic voltammograms of the PANI-based pH sensor with polymerization cycles ranging from 1 to 100 cycles. (**b**) The oxidative peak current (*I_peak_*, mA) of PANI-based GC electrodes with 25, 50, and 100 cycles at 0.2 V. (**c**) The logarithmic oxidative peak current (*logI_peak_*, µA) of PANI-based GC electrodes vs. PANI polymerization cycles (*N*). (**d**) *logI_peak_* vs. *logN*. (**e**) The corresponding cumulated charge *Q* of cyclic voltamograms of PANI polymerization at oxidative peak 0.2 V. (**f**) The cumulated charge *Q* of the corresponding cyclic voltammograms of PANI-based GC electrodes with 25, 50, and 100 cycles at 0.2 V with 50 mV/s. (**g**) The logarithmic charge (*logQ*, µC) of PANI-based GC electrodes vs. PANI polymerization cycles (*N*). (**h**) *logQ* vs. *logN.* Number of electrodes for PANI with 1–25, 25–50, and 50–100 cycles is 9, 6, and 3, respectively.

As shown in Figure 3b–d, the low-frequency capacitance at 10 mHz of the PANI-based GC electrode was enlarged as the solid-contact PANI film thickness increased from 25, 50, to 100 cycles [31]. The low-frequency capacitance at 10 mHz of the PANI-based GC electrode applied at 0.1 V, 0.15 V, and 0.2 V is obviously larger than 0 V, which is also in good agreement with cyclic voltammograms (Figure 4a), where the applied potential at ca. 0.2 V provides the oxidative peak of PANI, corresponding to the conductive proton state of emeralidine salt (ES). Low-frequency capacitance at 10 mHz of the PANI-based pH sensor with 25, 50, and 100 cycles increases accordingly at all applied potentials (Figure 3e,f, Table S1). Among all the applied potentials, the EIS with applied open circuit potential (OCP) gives the largest low-frequency capacitance, and the corresponding coulometric response of the PANI-based GC electrode with 50 cycles results in a large charge value with applied open circuit potential [33].

As shown in Table S2, the resistance of PANI 25, 50, and 100 cycles solid contact in 0.1 M HCl was decreased as the cycles of PANI increased with semicircle high frequency; the PANI resistance is dependent on the thickness of the solid contact and the electrolyte, independent of the applied potentials, which is also in good agreement with amperometric response of the current swift peak after potential changes (Figure 1c) [34,35]. The applied potentials have an impact on the low-frequency capacitance of the PANI-based pH sensor, while the resistance of the solid-contact PANI at a semicircle high frequency is independent of the applied potentials (Tables S1 and S2) [36,37].

The exponential growth behavior of the polyaniline (PANI) film is quantitatively corroborated through a comprehensive analysis of the cumulated faradaic charge (*Q*) associated with the oxidative peak at approximately 0.2 V (Figure 4) [38]. This current peak and the charge integrated from cyclic voltammograms provide a measure of the total electroactive material deposited. A bilinear relationship is revealed in the plot of logarithmic charge (*logQ*) versus the logarithmic number of polymerization cycles (*logN*), confirming two distinct growth regimes. The initial regime (1–25 cycles) was characterized by a power-law exponent (α) of 2.14, which is indicative of a super-linear, auto-catalytic growth phase. A subsequent transition leads to a second regime (30–100 cycles) with an even steeper exponent of 2.97 (Figure 4h). This progression is entirely consistent with the trends derived from the oxidative peak current analysis, where *logI_peak_* vs. *logN* polymerization cycles have slopes of 2.08 and 2.72, respectively (Figure 4d).

**Figure 5:**
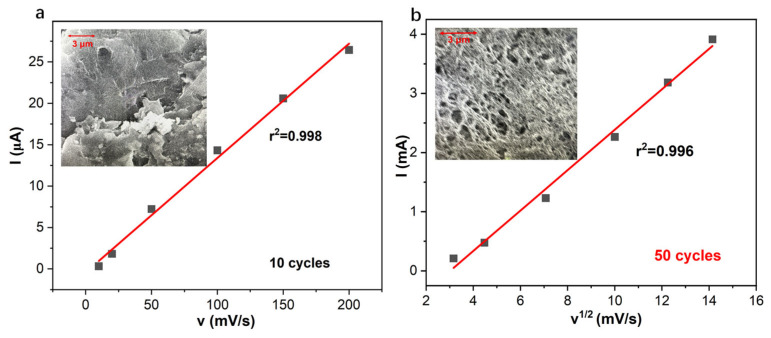
Oxidative peak current at approximately 0.2 V of the PANI-based pH sensor with 25 cycles and 50 cycles as a function of (**a**) scan rate and (**b**) square root of scan rate (50 cycles) in 1 M HCl.

The initial steep film growth, most pronounced within the first ~10 cycles (Figure 4c,g), exhibits slopes of ca. 0.13 µA/cycle and 0.13 µC/cycle for both *logIpeak* vs. *logN* and *logQ* vs. *logN,* which can be mechanistically attributed to highly efficient interfacial charge transfer at the pristine glassy carbon electrode surface. At the start-up stage, aniline monomers adsorb and undergo rapid nucleation and two-dimensional growth, leading to swift surface coverage and initial film thickening. The electrochemical signature of this mechanism is a linear dependence of the oxidative peak current on the scan rate (*v*) (Figure 5a), a hallmark of a surface-confined, adsorption-controlled process where the reaction rate is governed by the number of accessible surface sites [39,40].

As polymerization progresses beyond approximately 20−25 cycles, a fundamental kinetic transition occurs with the slopes decreasing to ca. 0.022 µA/cycle and 0.02 µC/cycle. The dominant growth mechanism shifts from lateral coverage to vertical thickening and the expansion of a complex internal surface area. This shift is decisively confirmed by the evolution of the scan rate dependence: the oxidative current transitions from a linear relationship with *v* to a linear relationship with *v*^0.5^, the classic signature of a diffusion-controlled process (Figure 5 and Figure S4).

The development of a porous, three-dimensional morphology at higher cycle numbers is consistent with this finding [40,41,42]. While this structure significantly increases the internal surface area, it also renders the polymerization rate dependent on the diffusion of aniline monomer and charge-compensating ions (e.g., Cl^−^) through the increasingly thick and tortuous film matrix. This mass transport limitation, coupled with incipient detrimental effects such as film peeling or oxidative degradation at advanced stages, contributes to the observed kinetic profile [43,44,45].

The relationship between the electrochemical signals and the physical growth of the film can be formalized according to Butler–Volmer kinetic equations:

(4) $$i=z_{i}FAk{\left[C_{s}\right]}^{1-\alpha }{\left[C_{p}\right]}^{\alpha }$$

where *z_i_* is the charge of the ion, *F* is the Faraday constant, *A* is the surface reaction area, *k* is the reaction constant, and *C_s_* and *C_p_* are the bulk concentration of the Cl^−^ in solution and aniline film, respectively. While the parameters are constant, the current is linearly proportional to the surface reaction area, and the faradaic charge *Q* is proportional to the electroactive surface area (*A*), which leads to the following:

*i* ∝ *A*, Q ∝ *A* (5)

The empirical power-law relationship (Figure 4h) is as follows:

*Q* *∝* *N^α^*, *A* *∝* *N^α^* (6)

where exponents α = 2.14 with 1–25 cycles PANI film and α = 2.97 at 30–100 cycles, and *N* denotes the number of polymerization cycles. For instance, during the first polymerization cycle, the quantity of aniline monomer consumed is approximately 2.7–4.8 × 10^10^ (Figure S3). This estimation is based on the assumptions that each aniline monomer polymerization involves the transfer of two electrons, the current efficiency during polymerization is 100%, and contributions from pseudocapacitance in the electrolyte are negligible [46,47].

This model of exponential area growth is powerfully supported by independent complementary techniques. Coulometric measurements of the PANI-based pH sensors reveal that the cumulated charge response per unit pH change surges from 2.49 µC/step for the 25-cycle film to 30 µC/step for the 50-cycle film. This represents an increase by a factor of over 12, which is substantially greater than the two-fold increase in cycle number, thereby validating the non-linear enhancement in charge storage capacity. Similarly, electrochemical impedance spectroscopy (EIS) provides direct insight into the interfacial properties. The low-frequency capacitance (*C_LF_*), a parameter directly proportional to the electroactive area, escalates from 182.8 µF for the 25-cycle film to 1065 µF for the 50-cycle film, a growth factor of 5.83. This significant expansion in interfacial capacitance aligns quantitatively with the expected increase in electroactive area predicted by the power-law model derived from the voltametric data.

The transition from a power-law exponent of 2.14 (1–25 cycles, indicative of 2D layer growth) to 2.97 (30–100 cycles, suggesting 3D porous growth (Figure 4 and Figure 5)) further elucidates the following: the thicker, porous films offer larger initial capacitance, resulting in longer pathways for proton and anion diffusion. This significantly slows down the redox kinetics, preventing complete re-protonation/reduction between measurements and predisposing the inner polymer layers to irreversible over-oxidation (Figure 1f), thereby degrading the sensor’s performance and long-term stability [2,23,29].

In summary, under the specified electrochemical conditions, the electrophysmerization of PANI is governed by an exponential growth law. The kinetics undergo a critical transition from an initial adsorption-controlled regime, characterized by rapid layer formation, to a subsequent diffusion-controlled regime responsible for developing a thicker, highly porous, three-dimensional architecture. The power-law exponents greater than 2 reveal a complex, non-linear growth process where the electroactive surface area expands at a rate far exceeding the linear addition of cycles, a fundamental insight crucial for the rational design of PANI films with tailored properties for advanced electrochemical applications, including the ion-to-electron transduction in SCISEs.

**Figure 6:**
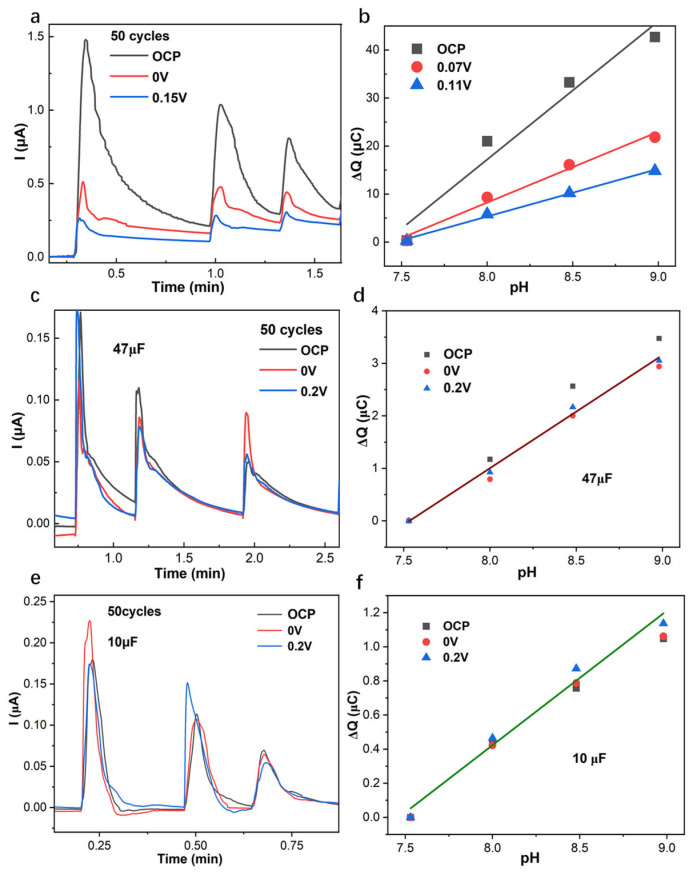
Amperometric response vs. time and the corresponding cumulated charge *Q* vs. pH of PANI-based GC electrode with 50 cycles at open circuit potential (OCP), 0 V and 0.2 V in the cases where there is no capacitor (**a**,**b**), and in series with capacitor 47 µF (**c**,**d**) and 10 µF (**e**,**f**).

The amperometric response (Figure 6a,c,e) of the PANI-based pH sensor with 50 cycles applied with OCP, 0 V, and 0.2 V with pH changes at 0.5 decades/step, and the linear response of the corresponding cumulated charge *Q* with respect to pH (Figure 6b,d,f) for the PANI-based GC electrode were demonstrated. As shown in Figure 6a,b, the amperometric and coulometric response of the PANI-based GC electrode demonstrates that applied open circuit potential gives larger current and cumulated charge than 0.07 V and 0.11 V, and also requires longer equilibrium time. The cumulated charge *Q* slope, with respect to pH for the PANI-based pH sensor without a capacitor, is influenced by the applied potentials OCP, 0 V, and 0.2 V, where the slope increases from 9.92 to 14.7 to 28.8 µC/pH. These results are in good agreement with the EIS low-frequency capacitance at 10 mHz (Table S1), where the EIS spectrum of the PANI electrode applied with open circuit potential gives a larger low-frequency capacitance (Figure 6b, Table S3). The linear slope of cumulated charge *Q* for the PANI-based pH sensor in series with capacitors 47 µF and 10 µF with respect to pH is unified and decreased to ca. average 2 µC/pH for 47 µF and 0.75 µC/pH for 10 µF in the case of pH changes at 0.5 decades/step. The resulting slope at variable potentials can be unified in connection with capacitors, which would be beneficial for the coulometric response to decrease the applied potential impact on the cumulated charge.

### 3.3. Application of Capacitive Coulometric Readout on Flexible PANI-Based pH Sensors

**Figure 7:**
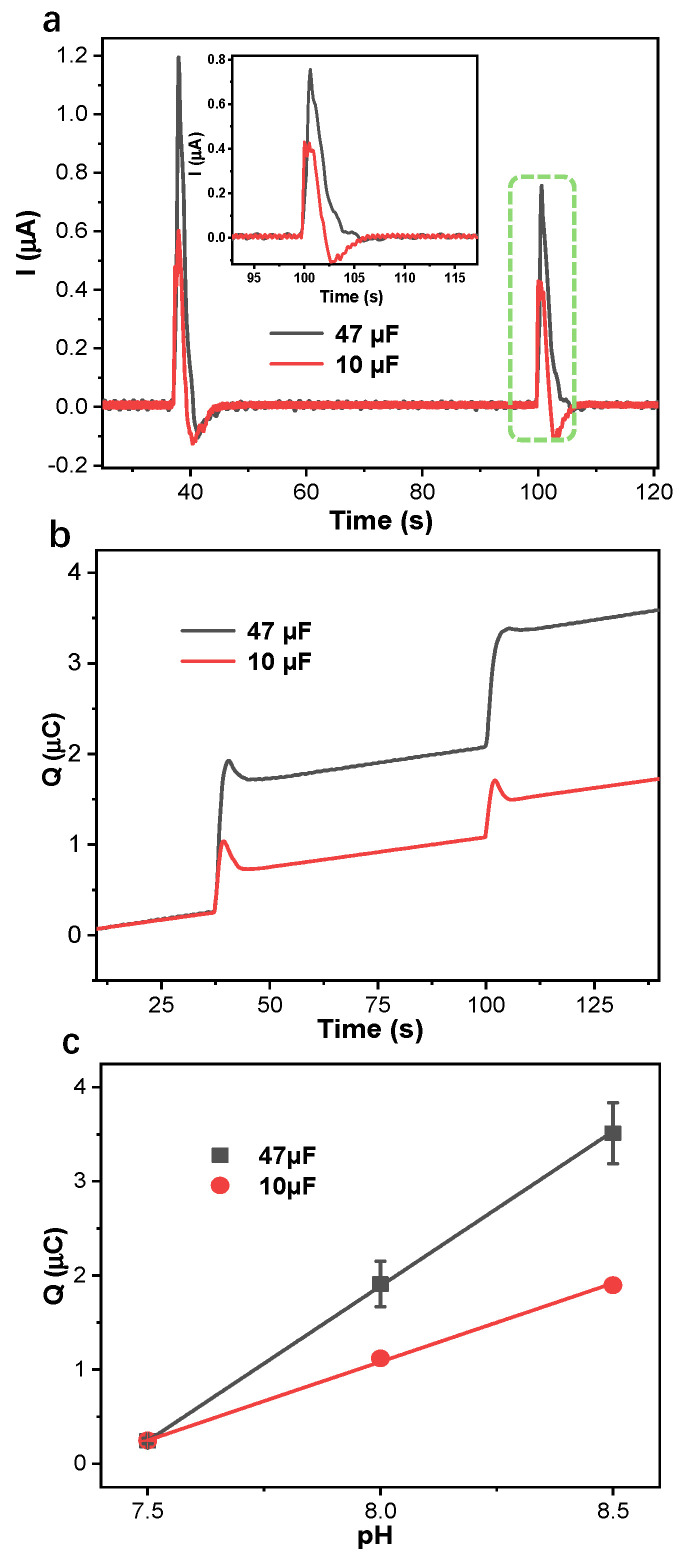
Amperometric (**a**) and coulometric (**b**) response of flexible PANI-based pH sensor (Փ_Au_ = 5 mm) with 50 cycles. The inset is the section enlargement of the amperometric response. (**c**) The linear cumulated charge *Q* of flexible PANI-based pH sensor vs. pH with error bar, when pH changes at 0.5 decades/step. Each parameter was measured with 3 electrodes.

The application of capacitive coulometric readout was further explored for the flexible PANI-based pH sensor (Figure 7). PANI solid contact was electropolymerized with 50 cycles on a flexible Au electrode surface. The potentiometric calibration of the PANI-based flexible sensors proves that the electrodes function well and follows the Nernst equation with a slope of 55 ± 2 mV/decade (Figure S2a,b).

As shown in Figure 7b,c, the amperometric and coulometric responses of flexible pH sensors with 50 cycles were performed with pH changes at 0.5 decades/step in series with capacitors 10 µF and 47 µF. While a flexible PANI-based pH sensor as a working electrode was in series with capacitors 10 µF and 47 µF, the response time can be limited to 5 s when pH changes at 0.5 decades/step. The connection with the capacitor 47 µF gives a larger cumulated charge *Q* than 10 µF, with the identical pH changes. And the linear slope of the cumulated charge *Q* of the flexible PANI-based pH sensor, with respect to pH changes, is 3.29 µC/pH for 47 µF and 1.67 µC/pH for 10 µF. This slope is larger than the PANI-based GC electrode (Փ_GC_ = 3 mm) because of the larger surface area of the flexible PANI-based pH sensor (Փ_AU_ = 5 mm), resulting in larger capacitance of PANI solid contact. Factors such as potential drift of the solid contact and the influence of variable electrolyte composition on the PANI redox kinetics could impact the stability and accuracy of the cumulated charge signal in such environments [29]. Nevertheless, this work establishes the significant potential of capacitive coulometric readout for flexible sensors, providing a foundation for future development towards on-body sweat sensors.

## 4. Conclusions

In this work, the amperometric and coulometric responses of PANI-based GC electrodes, fabricated with 25, 50, and 100 polymerization cycles, were systematically investigated for pH sensing. The study compared performance without a capacitor and in series with 10 µF and 47 µF capacitors. Without a capacitor, the coulometric response required a prolonged equilibrium time and often failed to reach a stable state within a practical duration. In contrast, the integration of an external capacitor significantly enhanced performance; under identical experimental conditions with pH changes of 0.5 decades/step, and the use of 47 µF and 10 µF capacitors, received cumulated charges *Q* of 2.02 μC and 0.8 μC, respectively. The principal advantage of the external capacitor is the drastic reduction in response time to a few seconds, which concurrently mitigates the detrimental impact of potential drift on the coulometric signal integrity.

Electrochemical impedance spectroscopy revealed that the high-frequency resistance of the PANI solid contact is governed by film thickness and electrolyte conductivity, remaining independent of applied potential. Conversely, the low-frequency capacitance (at 10 mHz) was significantly influenced by both the applied potential and the PANI film thickness. Analysis of the PANI film growth mechanism provided critical insights: the relationship between the deposited charge *Q* and the number of polymerization cycles followed a distinct power law. For 1–25 cycles, the exponent was approximately 2 (*logQ* vs. *logN* polymerization cycles slope of ~2.14), and a surface-controlled, two-dimensional (2D) layer-by-layer growth was observed. For 30–100 cycles, the exponent increased to approximately 3 (*logQ* vs. *logN* polymerization cycles slope of ~2.97), indicating a transition to a diffusion-controlled, three-dimensional (3D) growth mechanism, and resulting in a porous morphological structure, as observed at 50 cycles. This morphological evolution directly impacts ion transport kinetics, with thinner films (e.g., 10 cycles) being surface-controlled and thicker films (e.g., 50 cycles) becoming diffusion-limited.

The EIS measurements conducted at open-circuit potential revealed a large capacitance at 10 mHz. Similarly, constant potential coulometric responses obtained at open circuit potential exhibited a greater slope of accumulated charge with respect to an identical pH change. When in series with 10 µF or 47 µF capacitors, the linear response of cumulated charge *Q* of the PANI membrane-based GC electrodes with respect to pH is approximately 0.75 µC/pH and 2 µC/pH for pH changes of 0.5 decades/step, respectively. Furthermore, a preliminary study on the flexible PANI membrane-based pH sensors confirmed that constant potential coulometry is promising for use in wearable sensors for ion concentration detection.

## Data Availability

The data presented in this study are available on request from the corresponding author due to ongoing research.

## Supplementary Materials

The following supporting information can be downloaded at https://www.mdpi.com/article/10.3390/membranes15100320/s1, Figure S1: Cyclic voltammograms and the corresponding oxidative peak current at 0.2 V of polyaniline (PANI) with 25 (a,b), 50 (c,d), and 100 (e,f) cycles performed in 0.1 M aniline and 1 M HCl with a scan rate of 50 mV/s. Potential range is from −0.2 V to 0.82 V; Table S1: The EIS low-frequency capacitance at 10 mHz of the PANI-based pH sensor with applied potentials at open circuit potential (OCP), 0 V, 0.1 V, 0.15 V, and 0.2 V, and the oxidation peak current (*I_peak_*) at 0.2 V of cyclic voltammograms for the PANI-based pH sensor with 25, 50, and 100 cycles; Table S2: The PANI solid-contact resistance of the PANI-based pH sensor at semicircle high frequency, corresponding to real part *Z’* of electrochemical impedance spectrum; Table S3: The linear slope of the cumulated charge *Q* of the PANI-based pH sensor with 50 cycles with respect to pH without a capacitor, in series with capacitors 47 µF and 10 µF, when pH changes at 0.5 decades/step; Figure S2: Potentiometric measurement (a) and potentiometric calibration (b) of flexible PANI-based pH sensors. Amperometric (c) and coulometric (d) response of flexible PANI-based pH sensor. The pH range of the electrolyte was changed from 7, 7.5 to 8. The standard addition of pH changes was performed with the starting solution Tris-buffer +0.2 M NaOH by adding a certain volume of 0.2 M NaOH, resulting in pH changes at 0.5 decades/step. Figure S3: (a) The logarithmic oxidative peak current (*logI_peak_*, μA) of PANI-based GC electrodes vs. PANI polymerization cycles (*N*). (b) *logI_peak_* vs. *logN* with error bar (n = 3). (c) The logarithmic charge (*logQ*, µC) of PANI-based GC electrodes vs. PANI polymerization cycles (*N*). (d) *logQ* vs. *logN* with error bar (n = 3). Figure S4: Oxidative peak current at ca. 0.2 V of the PANI-based pH sensor with 25 cycles as a function of scan rate in 1.0 M HCl.
