# Supplementary material for: Capacitive Coulometric Readout of Polyaniline Membrane-Based pH Sensors in Combination with Cyclic Voltammetry and Electrochemical Impedance Spectroscopy
Source: Membranes (Basel). 2025 Oct 17;15(10):320. doi: 10.3390/membranes15100320 (PMC12565949; doi:10.3390/membranes15100320)
Supplement: Supplementary file 1 [file membranes-15-00320-s001.zip › membranes-3900318-supplementary.pdf]

Supporting information

Capacitive coulometric readout of polyaniline-based pH sensors in combination with cyclic voltammetry and electrochemical impedance spectroscopy

Tingting Han[a]*, Tao Song[b], Dongxue Han[a], Li Niu[a,c]

[1]Guangzhou Key Laboratory of Sensing Materials & Devices, Center for Advanced Analytical Science, School of Chemistry and Chemical Engineering, Guangzhou University, Guangzhou 510006, China; dxhan@gzhu.edu.cn (D.H.); lniu@gzhu.edu.cn (L.N.)

[2]State Key Laboratory of Pulp and Paper Engineering, South China University of Technology, Guangzhou 510640, China; songt@scut.edu.cn (T.S.)

[3]School of Chemical Engineering and Technology, Sun Yat-sen University, Zhuhai, 519082, China;

*Correspondence: tinghan@gzhu.edu.cn (T.H.)

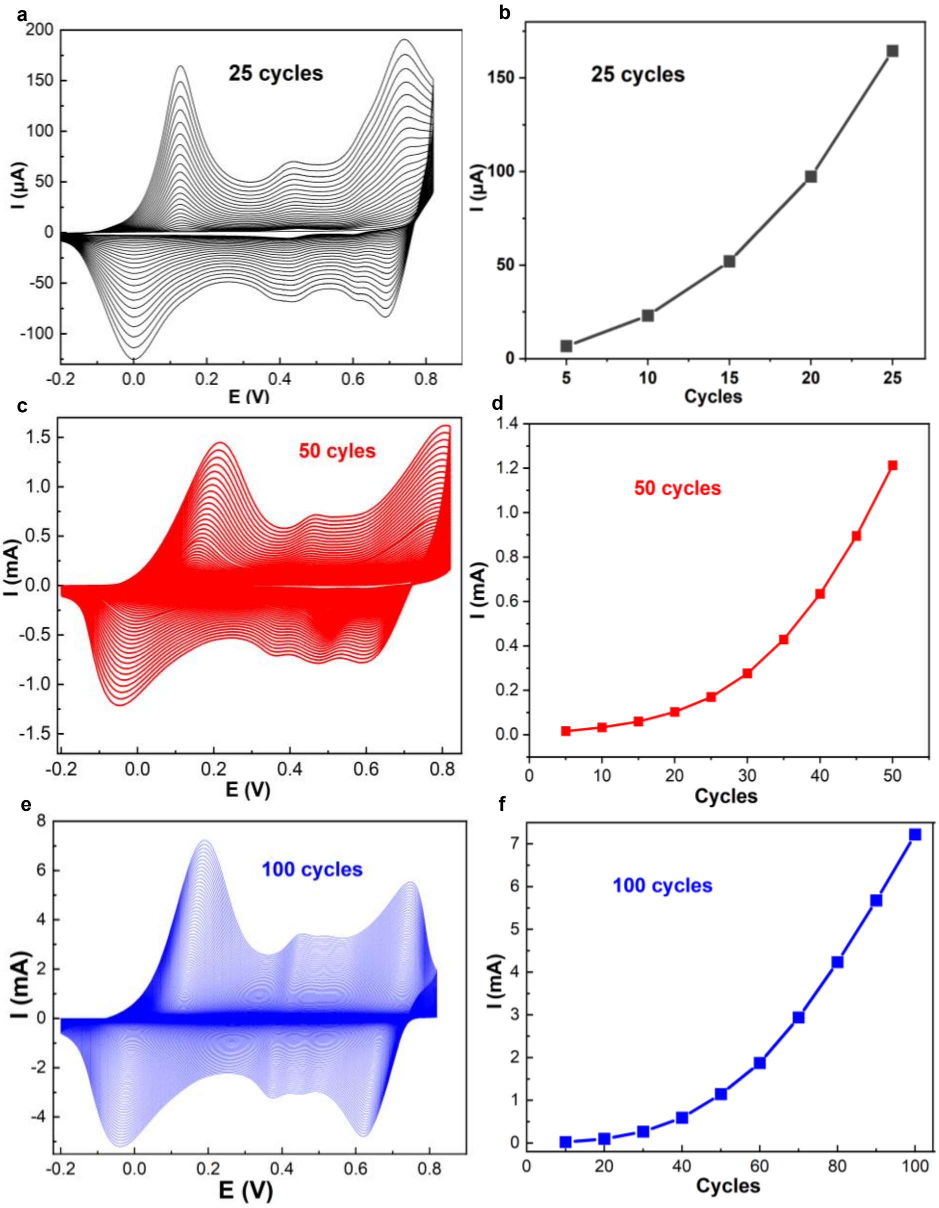

Figure S1. Cyclic voltammograms and the corresponding oxidative peak current at 0.2 V of polyaniline (PANI) with 25 (**a**, **b**), 50 (**c**, **d**), and 100 (**e**, **f**) cycles performed in 0.1 M aniline and 1 M HCl with a scan rate of 50 mV/s. Potential range is from -0.2 V to 0.82 V.

Table S1. The EIS low-frequency capacitance at 10 mHz of the PANI-based pH sensor with applied potentials at open circuit potential (OCP), 0 V, 0.1 V, 0.15 V, and 0.2 V, and the oxidation peak current (I_{peak}) at 0.2 V of cyclic voltammograms for the PANI-based pH sensor with 25, 50, and 100 cycles (Figure 3b, c ,d, e).

	**Low-frequency capacitance C_{LF} (µF) at 10 mHz**					**I_{peak} (µA)**
PANI	OCP	0 V	0.1 V	0.15 V	0.2 V	0.2 V
25 cycles	182.8	118.5	135.9	135.8	135.7	157.2
50 cycles	1065	702.1	794.3	781.6	770.8	1432
100 cycles	13130	8026	8991	8746	8461	7800

Table S2. The PANI solid contact resistance of the PANI-based pH sensor at semicircle high frequency, corresponding to real part Z' of electrochemical impedance spectrum.

	**PANI solid contact resistance Z' (Ω)**				
PANI	OCP	0 V	0.1 V	0.15 V	0.2 V
25 cycles	241.7	235.0	235.1	247.2	241.6
50 cycles	45.65	46.30	46.26	46.00	41.89
100 cycles	37.80	38.36	38.61	38.19	37.78

Table S3. The linear slope of the cumulated charge *Q* of the PANI-based pH sensor with 50 cycles with respect to pH without a capacitor, in series with a capacitor 47 μF and 10 μF, when pH changes at 0.5 decades/step.

Capacitor	***Linear slope of cumulated charge vs pH* (μC/pH)**			
(μF)	OCP	0 V	0.2 V	Average
without capacitor	28.8	14.7	9.92	Non-uniform
47 μF	1.84	2.08	2.15	2.02
10 μF	0.72	0.73	0.79	0.75

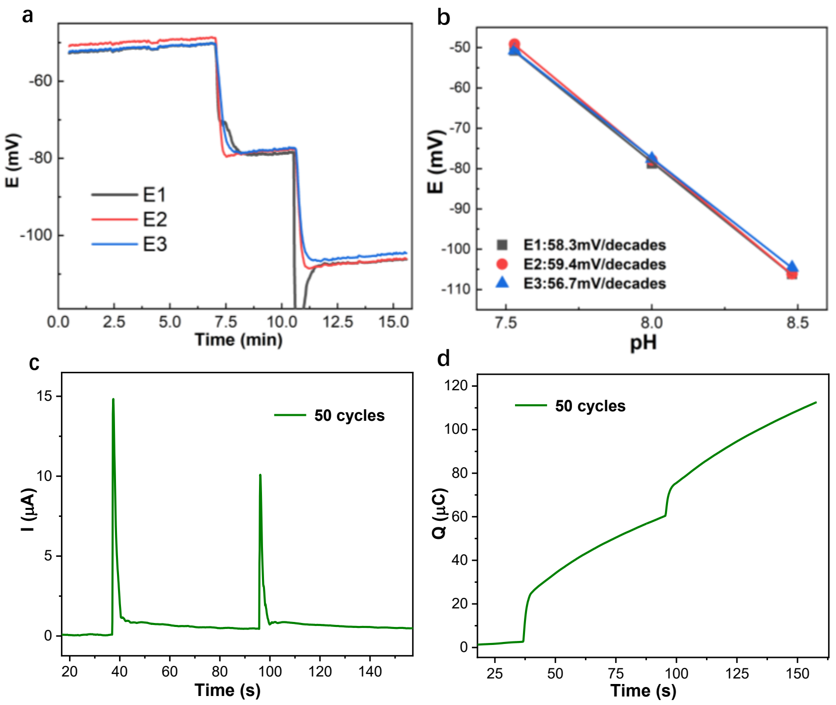

Figure S2. Potentiometric measurement (**a**) and potentiometric calibration (**b**) of flexible PANI-based pH sensors. Amperometric (**c**) and coulometric (**d**) response of flexible PANI-based pH sensor. The pH range of the electrolyte was changed from 7, 7.5 to 8. The standard addition of pH changes was performed with the starting solution Tris-buffer + 0.2 M NaOH by adding a certain volume of 0.2 M NaOH, resulting in pH changes at 0.5 decades/step.

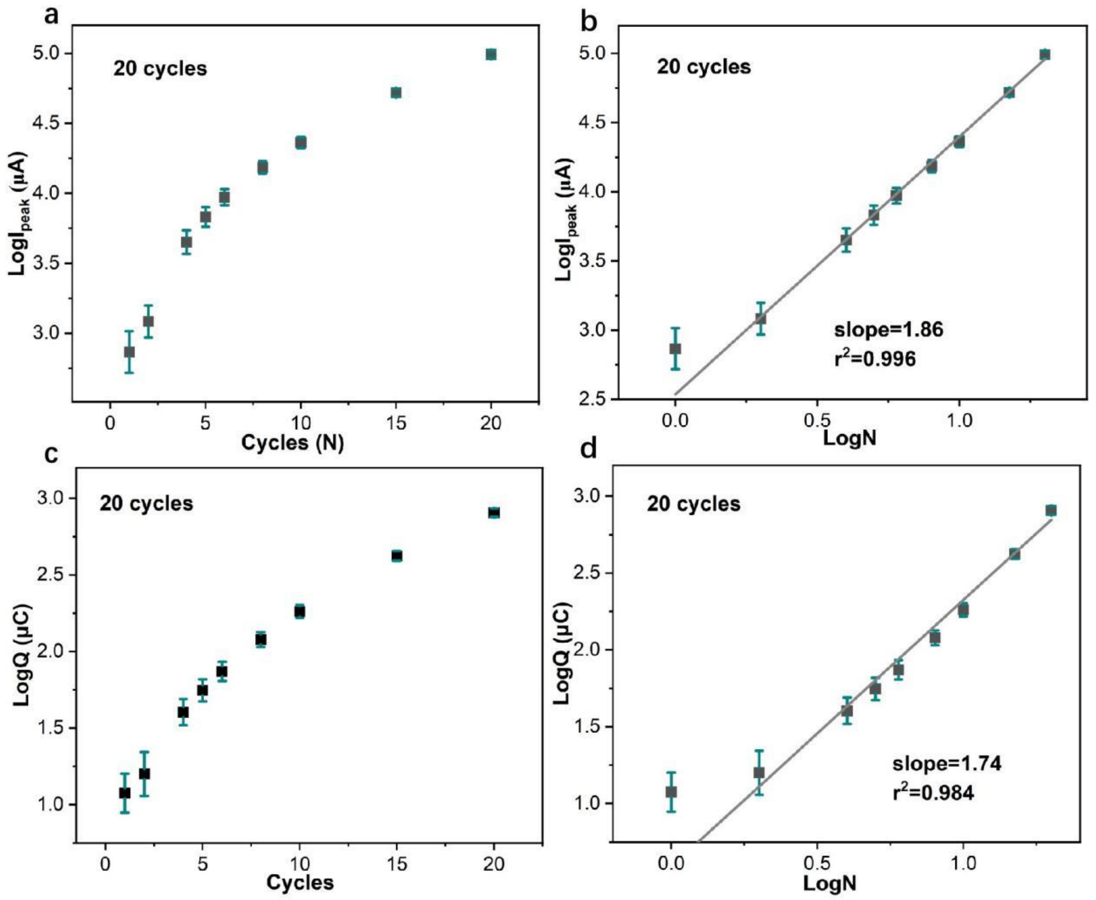

Figure S3. (**a**) The logarithmic oxidative peak current (*log*I_{peak}, μA) of PANI-based GC electrodes *vs* PANI polymerization cycles (*N*). (**b**) *log*I_{peak} *vs logN* with error bar (n=3). (**c**) The logarithmic charge (*logQ*, μC) of PANI-based GC electrodes *vs* PANI polymerization cycles (*N*). (**d**) *logQ vs logN* with error bar (n=3).

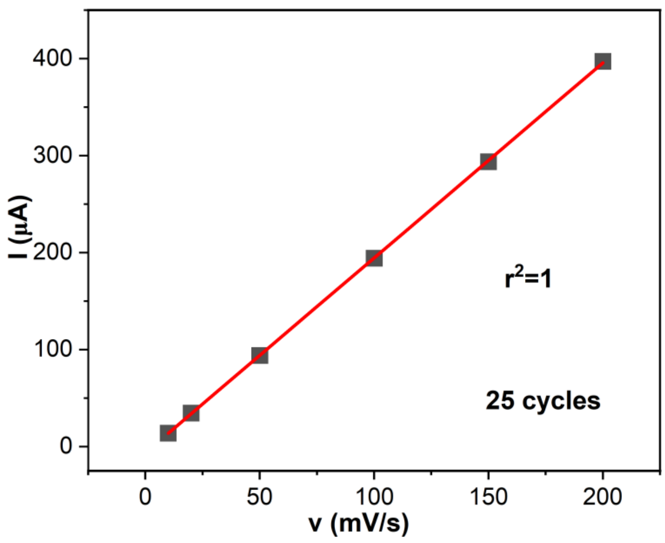

Figure S4. Oxidative peak current at approximately 0.2 V of the PANI-based pH sensor with 25 cycles as a function of scan rate in 1.0 M HCl.
